# Comparative evaluation of LDL-cholesterol estimation using machine learning algorithms and conventional equations in a large middle eastern cohort

**DOI:** 10.1016/j.clinsp.2026.101068

**Published:** 2026-09-08

**Authors:** Fayha Salah Ahmed, Mohammad Imteyaz Ahmad, Mohamedi Begum, Deepa Rao, Sheifa Joan

**Affiliations:** a College of Medicine, Mohammed Bin Rashid University of Medicine and Health Sciences, Dubai Health, Dubai, United Arab Emirates; b Clinical Chemistry Immunology Division, Dubai health, Dubai, United Arab Emirates

**Keywords:** Triglycerides, LDL-C estimation, Machine learning, Cardiovascular risk, Hypertriglycerides

## Abstract

**Background:**

Accurate estimation of Low-Density Lipoprotein Cholesterol (LDL-C) is critical for cardiovascular risk assessment. Traditional equations like Friedewald often underperform, especially in patients with high triglyceride levels. This study compares the performance of Machine Learning (ML) algorithms with conventional equations for LDL-C estimation.

**Methods:**

A retrospective analysis was conducted using 96,492 lipid profiles from Dubai Health. LDL-C was directly measured for triglycerides > 400 mg/dL and estimated using Friedewald, Martin-Hopkins, and Sampson equations for TG < 400 mg/dL. ML models, Random Forest, XGBoost, Neural Network, k-Nearest Neighbors (k-NN), and Bayesian Ridge, were trained using total cholesterol, HDL-C, and triglycerides as predictors.

**Results:**

Machine learning models demonstrated higher statistical agreement with directly measured LDL-C than conventional equations in patients with triglyceride levels < 400 mg/dL. Model performance declined substantially in hypertriglyceridemia subgroups (> 400 mg/dL), with increased prediction error across all approaches.

**Conclusion:**

Machine learning–based LDL-C estimation shows improved agreement with routinely reported direct LDL-C values under specific triglyceride conditions. However, performance limitations in hypertriglyceridemia and the absence of external validation indicate that ML approaches should be considered complementary rather than replacement methods.

## Highlights

• Machine learning models improved LDL-C estimation versus conventional equations.
• Random Forest and Bayesian Ridge showed highest agreement at TG < 400 mg/dL.
• Prediction accuracy declined markedly in hypertriglyceridaemic patients.
• ML-based LDL-C estimation should complement, not replace, existing methods.

## Introduction

Low Density Lipoprotein Cholesterol (LDL-C) is a key biomarker in cardiovascular medicine due to which it serves as a primary therapeutic target in primary and secondary prevention of Atherosclerotic Disease (ASCVD). Due to this importance accurate measuring of LDL-C is essential for the purpose of diagnosis, risk stratification and monitoring of treatment.^1^ The clinical guidelines consistently emphasize on the importance of LDL-C reduction for lowering cardiovascular risk while highlighting the need of reliable methods of measurements and estimation.^1^ Moreover, LDL-C has been measured by laboratory assessments or with utilization of formulas for calculation. For instance, The Friedewald equation is a commonly applied formula for measuring parameters like cholesterol, triglycerides and high-density lipoprotein cholesterol.^2^ This equation was introduced in 1972 and it is applied in practice due to its simplicity and reliance on routinely measured parameters.^2^ However, despite the Friedewald equation being widely used in practice, it demonstrates reduced accuracy when LDL-C levels are low and in the presence of elevated triglycerides. Therefore, estimation of LDL-C in patients with low levels can result in inappropriate classification of risks and mismanagement of the suggested therapy.^2^ In order to overcome these limitations of Friedewald equation new estimation equations were proposed such as Martin-Hopkins equation which introduced adjustable factor for measuring Low Density Lipoprotein Cholesterol (VLDL-C), that is derived from triglyceride and non-HDL-C ratios in different strata due to which accuracy in wide lipid spectrum is improved.^3^ At the same time, Sampson et al. also developed regression-based equation by using a large database of lipid profiles gathered from the National Institutes of Health and has shown enhanced accuracy and precision and reduced bias as compared to the Friedwald equation.^4^ Therefore, these equations as an alternative of Friedwald equation have improved performance but inaccuracies are still pertaining specifically in dyslipidemia populations.^5^, ^6^, ^7^

On the other hand, the development of refined formulas and advancements in artificial intelligence have created opportunities for data driven modeling of Lipid parameters. At the same time, Machine Learning techniques are also playing an important role in measuring complex nonlinear relationships in clinical and biochemical variables, outperforming conventional linear equations in LDL-C estimation.^8^^,^^9^ Thus, several machine learning algorithms like Random Forest, gradient boosting (XGBoost), neural networks, k-Nearest Neighbors (k-NN) and Bayesian regression are also explored for their biomedical applications and supporting for enhancing laboratory medicine.^8^, ^9^, ^10^ In previous studies, it has been suggested that machine learning models can provide superior LDL-C estimation as compared to traditional equations in specific clinical subgroups.^9^^,^^11^ In this regard, XGBoost has demonstrated improved accuracy in hyper triglyceridemic patients and Random Forest models have shown high correlation with measured LDL-C.^9^^,^^12^ Similarly, neural networks despite being powerful, require extensive optimization and large datasets for achieving reliable results.^8^ Whereas Bayesian approaches provide probabilistic predictions for adding value by quantifying uncertainty in clinical decision making.^10^

Despite presenting strong findings, recent studies have been limited by small sample sizes, lack of diverse population and narrow focus on selected algorithms. At the same time, direct comparisons of conventional formulas and machine learning algorithms in large datasets is also scarce.^9^^,^^11^ Thus, indicating a critical gap for validation of data in different patient populations before machine learning algorithms are integrated in clinical practices. Therefore, the study was designed to conduct large scale comparative evaluation of LDL-C estimation by using conventional calculation methods like Friedwald, Martin-Hopkins and Sampson and machine learning algorithms. For this purpose, the authors aimed to:

• Assess the accuracy and precision of conventional formulas when compared with machine learning models by using direct LDL-C measurements as standards.
• Examine performance of subgroups based on triglyceride levels (> 400 mg/dL vs. < 400 mg/dL) and sex.
• Evaluate classifications according to National Cholesterol Education Program (NCEP) Adult Treatment Panel III categories.

Through these objectives, the authors seek to compare the performance of machine learning-based LDL-C estimation methods with conventional equations under routine laboratory conditions, while evaluating their strengths and limitations across triglyceride subgroups.

### Materials and methods

The study follows the STROBE checklists guidelines for retrospective cross-sectional study. The study was conducted in the Department of Clinical chemistry and Immunology, Dubai Health, and included lipid profiles that were performed between January 2022, and December 2022. For this purpose, inpatient and outpatient samples were included in the study, whereas ethical approval for the study was taken from the Institutional Review Board of Mohammad Bin Rashid University of Medical and Health Sciences. At the same time, data was extracted from centralized laboratory information system of Dubai Health. As a result, a total of 96,492 lipid profiles were retrieved from the information system where each profile contained measurements of cholesterol, triglycerides, High Density Lipoprotein Cholesterol (HDL-C) and Low-Density Lipoprotein Cholesterol (LDL-C). Moreover, the records that were with missing values or had results outside linearity of the assays were excluded and for preventing duplication of data first record of lipid profile was considered for analysis especially where a patient had multiple lipid profiles. As a result, final dataset contained 55,151 female lipid profiles and 41,341 male lipid profiles. The dataset included adult patients across a wide age range (18–85 years), representing routine outpatient and inpatient testing populations. Both fasting and non-fasting lipid profiles were included, reflecting real-world laboratory practice. Fasting status was not consistently recorded in the laboratory information system and therefore formal stratification by fasting versus non-fasting state was not feasible. As a result, analyses were conducted on the combined dataset to reflect routine clinical practice. This approach may have introduced additional biological variability, particularly affecting triglyceride levels and LDL-C estimation accuracy, and this limitation has been considered when interpreting subgroup performance. Major clinical subgroups such as diabetes, hypertension, and dyslipidemia were not excluded, as the study aimed to capture the full clinical spectrum of patients undergoing lipid testing. Information on specific clinical conditions such as diabetes mellitus, hypertension, and diagnosed dyslipidemia was not uniformly available in the extracted dataset and therefore could not be included as covariates in the analysis. Consequently, no adjustment for these clinical confounders was performed. This may have influenced LDL-C estimation performance, particularly in subgroups with altered lipid metabolism, and represents a limitation of the study. Patient-level deduplication was performed by retaining only the first lipid profile recorded for individuals who had multiple tests during the study period to prevent duplicate representation in the analysis. Moreover, all biochemical measurements were performed in accredited Dubai Health Laboratories by using Roche Cobas automated chemistry analyzers (Roche Diagnostics, Mannheim, Germany), and the total cholesterol was determined by enzymatic cholesterol oxidase/peroxidase method, triglycerides were measured by the enzymatic glycerol phosphate oxidase/peroxidase method. At the same time, HDL-C was assessed by direct homogeneous tests and LDL-C was measured by utilizing a homogeneous detergent-based tests in patients with triglyceride levels above 400 mg/dL. For this study, the reference LDL-C differed by triglyceride subgroup. Although ultracentrifugation-based beta-quantification is considered the theoretical reference method for LDL-C measurement, it is not routinely available in clinical laboratories. In this study, directly measured LDL-C obtained using a homogeneous detergent-based assay on the Roche Cobas platform was used as a clinical comparator to reflect real-world laboratory practice rather than as a gold-standard reference. Accordingly, the results should be interpreted as relative agreement and comparative performance against a routinely used direct assay, rather than absolute accuracy against beta-quantification.

For TG > 400 mg/dL, the reference was the directly measured LDL-C obtained using the Roche homogeneous detergent-based assay. For TG < 400 mg/dL, the reference was the directly measured LDL-C available in the laboratory information system, against which the Friedewald, Martin-Hopkins, Sampson, and ML-predicted LDL-C values were compared. Although beta-quantification is the historical ultracentrifugation-based gold standard, it is not routinely performed in clinical laboratories; therefore, direct LDL-C served as the practical reference standard for this analysis. The direct LDL-C assay used in Dubai Health laboratories is a homogeneous, enzymatic, detergent-based method performed on the Roche Cobas platform. The assay is traceable to CDC reference materials, with manufacturer-reported total imprecision typically < 3% across the analytical measuring range. The method meets the National Cholesterol Education Program (NCEP) allowable total error goal of ±12%. As with all homogeneous direct LDL-C assays, potential limitations exist in cases of marked hypertriglyceridemia, where interference from triglyceride-rich lipoproteins may affect specificity. It is also noted that direct LDL-C methods do not represent the historical ultracentrifugation-based beta-quantification reference method, which remains the theoretical gold standard but is not routinely available in clinical practice. Further, patients with triglyceride concentrations below 400 mg/dL, LDL-C was estimated using three different calculation methods like the Friedewald equation,^2^ Martin-Hopkins adjustable factor equation,^3^ and the Sampson regression-based equation developed at the NIH.^4^ These analyses were performed with Roche reagents and calibrators, and internal quality assurance was measured through use of Precicontrol quality control materials. Internal Quality Control (IQC) was performed twice daily using Precicontrol Normal and Precicontrol Pathological QC materials (Roche Diagnostics). QC target values were maintained within manufacturer-recommended ranges throughout the study period, and Westgard-based QC rules were applied according to routine laboratory practice. No major reagent lot or calibrator changes occurred during the study period that would have materially affected the accuracy or precision of LDL-C, HDL-C, triglycerides, or total cholesterol measurements. All assays operated within allowable imprecision limits defined by NCEP and manufacturer specifications. In addition to conventional calculation methods, machine learning algorithms were developed for predicting LDL-C values, for which model development was carried using Scikit-learn library in python (version 3.8). At the same time, predictor variables included total cholesterol, triglycerides and HDL-C, while LDL-C was used as dependent variable. In order to minimize the risk of data leakage, patient-level deduplication was completed prior to model development, ensuring that no individual contributed records to both training and testing subsets. Patient-level deduplication was performed before any data partitioning. Following deduplication, the dataset was randomly divided into training (70%) and testing (30%) subsets at the patient level, ensuring that no individual contributed data to both sets. This workflow prevented information leakage between training and testing datasets and reduced the risk of overoptimistic model performance estimates. Outliers beyond the analytical linearity limits of the assays were removed before model fitting. Hyperparameter optimization for all machine learning algorithms was performed using ten-fold cross-validation within the training set. The final models were evaluated exclusively on the held out 30% test dataset. External validation using an independent temporal or geographically distinct cohort was not performed due to the absence of an additional institutional dataset available for linkage. Consequently, model performance reflects internal validation only. While the large sample size supports internal stability, external validation is required before generalizability and clinical deployment can be established. For the TG < 400 mg/dL subgroup, the reference LDL-C value reported by the laboratory is itself calculated using the Friedwald equation. Therefore, the extremely high R^2^ values achieved by ML models in this subgroup reflect their ability to learn this fixed mathematical relationship, not an improvement in biological accuracy. To mitigate overfitting, hyperparameter optimization for all models was performed using ten-fold cross-validation restricted to the training dataset, and model performance was reported only on the independent test dataset. No feature selection was performed using outcome information, and no individual contributed data to both training and testing sets.^5^^,^^7^^,^^9^ For triglyceride-based subgroup analysis, patients were stratified using a threshold of 400 mg/dL, consistent with current clinical practice in which calculated LDL-C equations are considered unreliable above this level. While this dichotomous stratification reflects routine laboratory decision thresholds, it does not capture intermediate triglyceride ranges (e.g., < 150 mg/dL and 150–399 mg/dL), which may exhibit differential estimation performance. This limitation has been considered in the interpretation of results. For sex-based stratification, individual analysis was performed for males and females and in the subgroups with triglycerides greater than 400 mg/dL, machine learning model predictions were compared with directly measured LDL-C values. Sex-based analyses were conducted as stratified descriptive comparisons. No multivariable regression modelling or interaction analysis was performed to evaluate sex as an independent predictor of LDL-C estimation performance after adjustment for triglyceride levels or other clinical factors. Accordingly, observed differences between males and females should be interpreted as descriptive rather than causal. Whereas in the subgroups with triglycerides below 400 mg/dL, predications of machine learning models were compared with LDL-C values that were calculated using the Friedwald, Martin-Hopkins and Sampson equations.

The R software (version 4.2) and Python Scikit-Learn were used for statistical analysis and the descriptive statistics including mean, standard deviation, skewness, kurtosis, percentiles and 95% Confidence Intervals were separately identified for male and female patient populations. At the same time, performance of model was evaluated using the coefficient of determination (R^2^), mean absolute error and root mean square error, while for assessing clinical application, LDL-C results were further classified as per the guidelines of National Cholesterol Education Program Adult Treatment Panel III into optimal, near optimal, borderline high, high and very high with the values ranging between < 100 mg/dL, 100‒129 mg/dL, 130‒159 mg/dL, 160‒189 mg/dL and ≥ 190 mg/dL respectively. Additionally, agreement between methods was assessed by using Cohen’s Kappa statistics and confusion matrix analysis.^6^^,^^9^^,^^11^

## Results

**Fig. 1:**
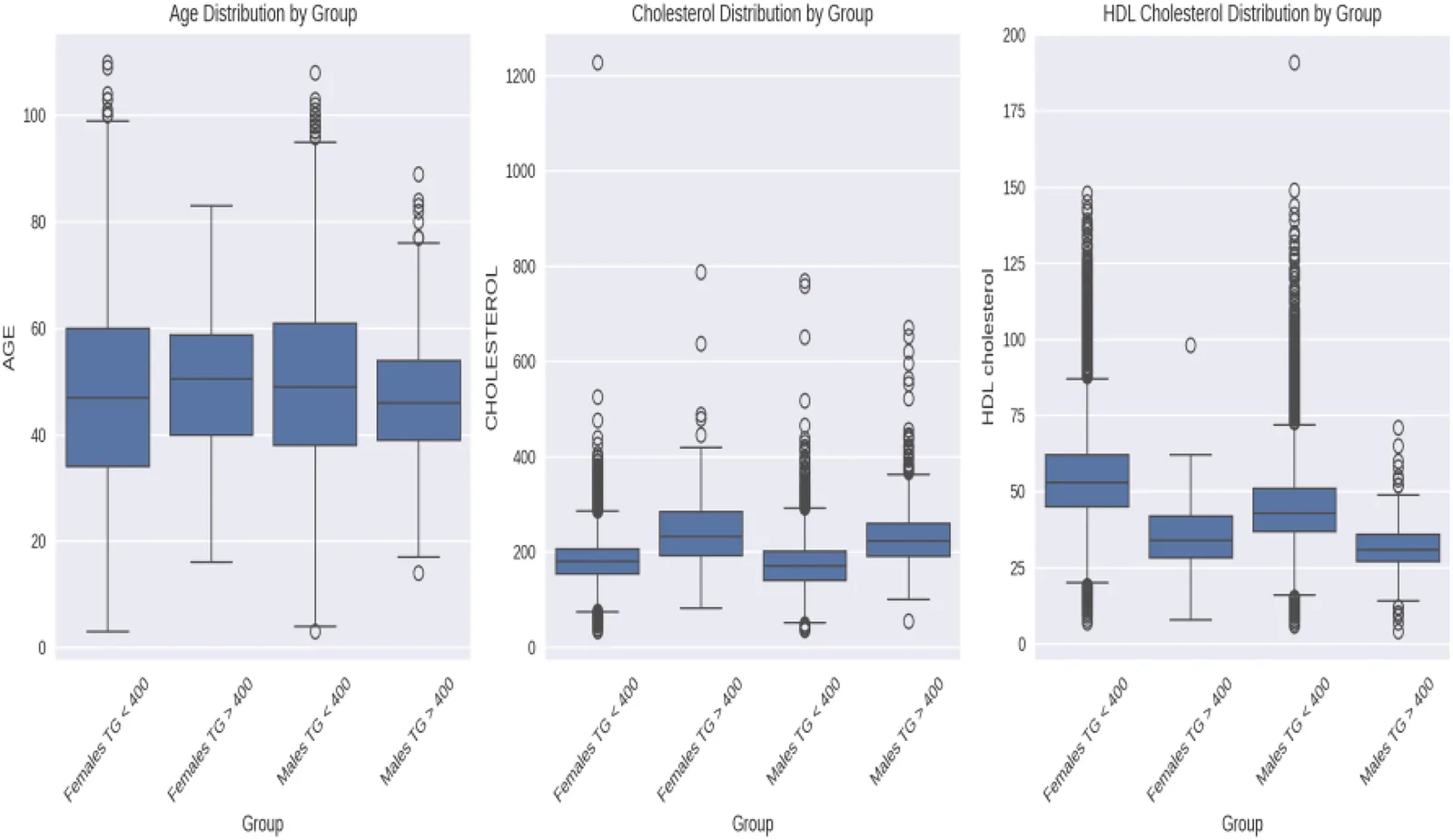
Distribution of age, total cholesterol, HDL-C, stratified by triglyceride group and sex.

**Fig. 2:**
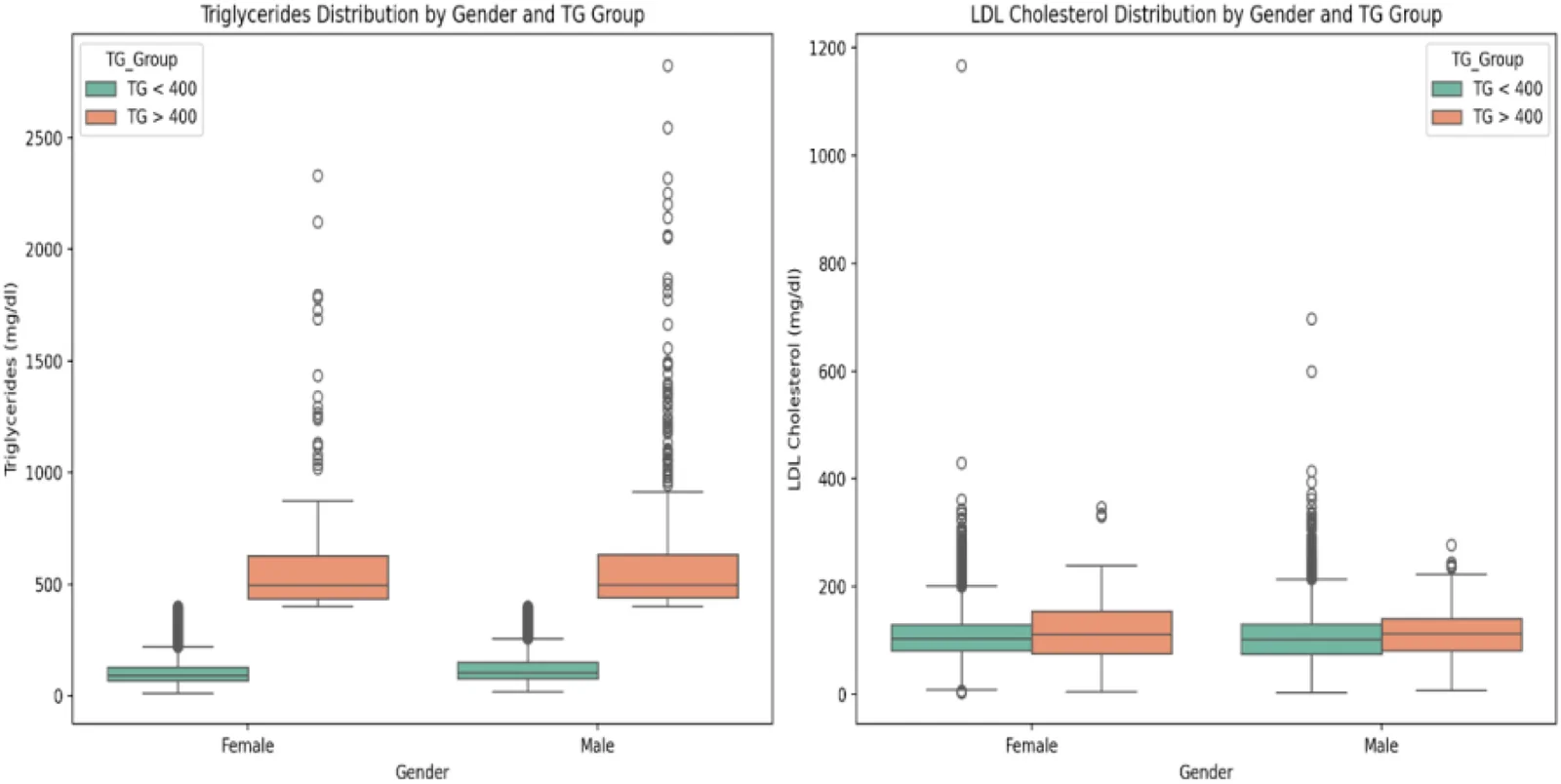
Distribution of Triglycerides, LDL-C, stratified by triglyceride group and sex.

The dataset was based on 96,492 lipid profiles from which 55,551 were females and 41,341 males, where the descriptive characteristics of lipid parameters in females and males are summarized in Table 1, Table 2. Fig. 1 present the distribution of age, total cholesterol, HDL-C, stratified by triglyceride groups (< 400 mg/dL and > 400 mg/dL) to illustrate data distribution and variability across subgroups. Within this subgroup, total cholesterol distributions were relatively symmetrical across sexes, whereas females exhibited higher HDL-C levels (mean 54.4 mg/dL vs. 44.7 mg/dL in males). Fig. 2 presents the distribution of triglycerides and LDL-C across triglyceride-defined subgroups and sex. Triglycerides displayed a markedly skewed distribution, particularly in males with extreme values reaching up to 2824 mg/dL in males and 2330 mg/dL in females. LDL-C distributions were relatively symmetrical across sexes.

**Table 1:** Descriptive statistics of females (All lipid values are reported in mg/dL).

Statistics	Total Cholesterol	HDL cholesterol	Triglycerides	NHDL Cholesterol
Total	55,151	55,151	55,151	55,151
Mean	182.3	54.4	107.0	127.9
SD	40.2	13.8	63.8	39.0
Maximum	1228	148	2330	1221
Minimum	33	7	15	14
kurtosis	10.4	1.3	106.7	14.1
skewness	0.9	0.7	5.7	1.2
CI lower	182	54	106	128
CI upper	183	55	108	128
5th percentile	122	35	45	72
95th percentile	251	79	213	196

**Table 2:** Descriptive statistics of males (All lipid values are reported in mg/dL).

Statistics	Total Cholesterol	HDL cholesterol	Triglycerides	NHDL Cholesterol
Total	41,341	41,341	41,341	41,341
Mean	174.5	44.7	129.7	129.8
SD	45.1	11.7	95.6	44.7
Maximum	769	191	2824	756
Minimum	36	4	21	16
kurtosis	3.6	3.8	95.8	4.4
skewness	0.7	1.1	6.5	0.9
CI lower	174	45	129	129
CI upper	175	45	131	130
5th percentile	108	29	50	66
95th percentile	250	66	275	206

**Fig. 3:**
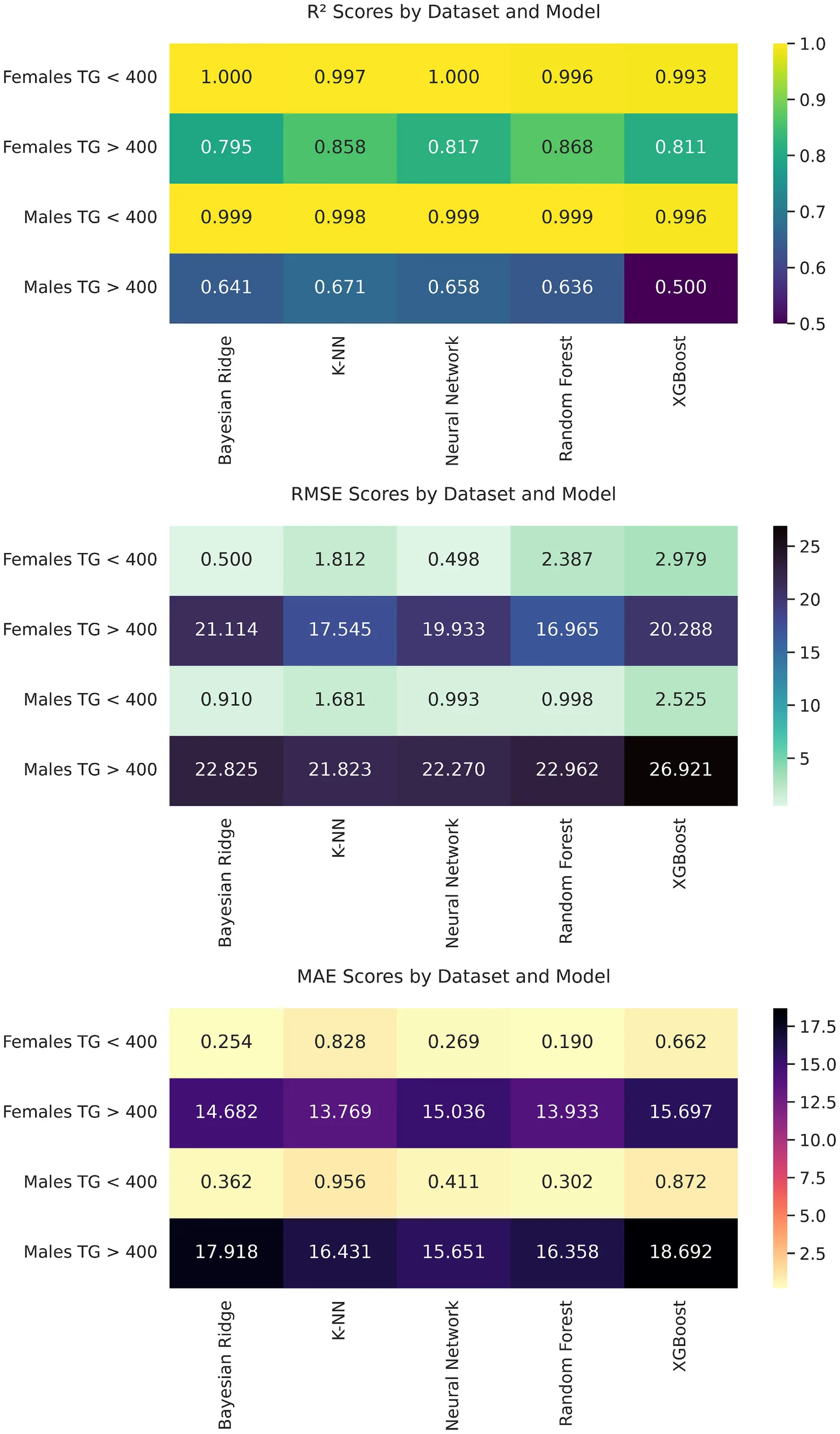
Comparison of machine learning models based on R^2^, RMSE and MAE.

Fig. 3 compares machine learning model performance using R^2^, RMSE, and MAE across triglyceride strata. In the TG < 400 mg/dL subgroup, all models achieved excellent agreement with reference LDL-C, with R^2^ values ranging from 0.996 to 1.000 in females and 0.996 to 0.999 in males. Correspondingly, prediction errors were minimal (MAE ≤ 0.96 mg/dL across models).

In contrast, model performance declined substantially in the TG > 400 mg/dL subgroup, with R^2^ values decreasing to 0.795–0.868 in females and 0.500–0.671 in males. This deterioration was accompanied by increased RMSE (up to 26.92 mg/dL) and MAE (up to 18.69 mg/dL), indicating reduced predictive accuracy. Random Forest demonstrated relatively better performance in this subgroup; however, no model maintained consistently high accuracy.

Table 3 represents demographic characteristics of full cohort and patient characteristics included in the study. Table 4 In the TG < 400 mg/dL group, most machine learning models demonstrated very high agreement with directly measured LDL-C, reflected by R^2^ values approaching 1.00 and low absolute error. In this subgroup, Random Forest and Bayesian Ridge models showed consistently low MAE across both sexes. Although Neural Network models also demonstrated high statistical performance metrics in TG < 400 mg/dL, their performance was less stable across subgroups and declined in TG > 400 mg/dL. In patients with TG > 400 mg/dL, all models showed reduced performance, with k-nearest neighbors and Random Forest models demonstrating comparatively lower error than other approaches. These findings indicate that while several ML models perform well under low triglyceride conditions, their reliability decreases in hypertriglyceridemia.^9^, ^10^, ^11^

**Table 3:** Baseline demographic and clinical characteristics of the study population.

Characteristic	Value
Total lipid profiles included	96,492
Females, n (%)	55,151 (57.1%)
Males, n (%)	41,341 (42.8%)
Age range	18–85 years (detailed age distribution not available)
Fasting status	Not consistently recorded in LIS; includes both fasting and non-fasting samples
Major clinical subgroups (diabetes, hypertension, dyslipidemia)	Not available in extracted dataset
Patient-level deduplication	Yes, only first lipid profile per patient was retained
Study setting	Dubai Health outpatient and inpatient services

**Table 4:** Machine learning prediction model for females and males with TG > 400 mg/dL and TG < 400 mg/dL.

Dataset	Model	R^2^	RMSE	MAE
Females TG < 400	Random Forest	0.996	2.39	0.19
Females TG < 400	XGBoost	0.993	2.98	0.66
Females TG < 400	Neural Network	1.000	0.50	0.27
Females TG < 400	K-NN	0.997	1.81	0.83
Females TG < 400	Bayesian Ridge	1.000	0.50	0.25
Females TG > 400	Random Forest	0.868	16.96	13.93
Females TG > 400	XGBoost	0.811	20.29	15.70
Females TG > 400	Neural Network	0.817	19.93	15.04
Females TG > 400	K-NN	0.858	17.54	13.77
Females TG > 400	Bayesian Ridge	0.795	21.11	14.68
Males TG < 400	Random Forest	0.999	1.00	0.30
Males TG < 400	XGBoost	0.996	2.52	0.87
Males TG < 400	Neural Network	0.999	0.99	0.41
Males TG < 400	K-NN	0.998	1.68	0.96
Males TG < 400	Bayesian Ridge	0.999	0.91	0.36
Males TG > 400	Random Forest	0.636	22.96	16.36
Males TG > 400	XGBoost	0.500	26.92	18.69
Males TG > 400	Neural Network	0.658	22.27	15.65
Males TG > 400	K-NN	0.671	21.82	16.43
Males TG > 400	Bayesian Ridge	0.641	22.82	17.92

* Subgroup sample sizes (n = 54501 females) (n = 40,691 males) for TG < 400 mg/dL and TG > 400 mg/dL (n = 650 Females) (n = 650 males) in males and females However, the distribution of profiles across these strata reflects the overall sex proportions in the study population, and subgroup analyses were performed accordingly.

Table 4 The exceptionally high R^2^ values observed for Neural Network and Bayesian Ridge models in the TG < 400 mg/dL subgroups reflect the constrained biochemical variability and strong deterministic relationships among lipid parameters within this range, rather than perfect physiological prediction. Despite the use of an independent test dataset and cross-validation during training, these metrics should be interpreted cautiously as indicators of statistical fit rather than generalizable clinical accuracy. Importantly, Neural Network performance declined substantially in TG > 400 mg/dL subgroups, indicating reduced robustness under conditions of greater lipid heterogeneity. This may be due to high interferences with direct LDL cholesterol data of the laboratory leading to high variability in training dataset leading to high variability in output of ML models. Table 5 represents mean performance metrics for males and females for R^2^, RMSE and MAE.

**Table 5:** Mean performance metrics of all dataset.

Model	R^2^	RMSE	MAE
Females TG < 400	0.997	1.63	0.44
Females TG > 400	0.829	19.16	14.62
Males TG < 400	0.998	1.42	0.5
Males TG > 400	0.612	23.36	17

Model performance was substantially higher in TG < 400 mg/dL compared with TG > 400 mg/dL. In lower triglyceride strata, Random Forest and Bayesian Ridge consistently demonstrated lower MAE and higher agreement across sexes. In hypertriglyceridemia subgroups, prediction error increased for all models, with no single approach demonstrating robust performance. These findings indicate that apparent model superiority in low triglyceride conditions does not extend uniformly to higher triglyceride ranges.

**Fig. 4:**
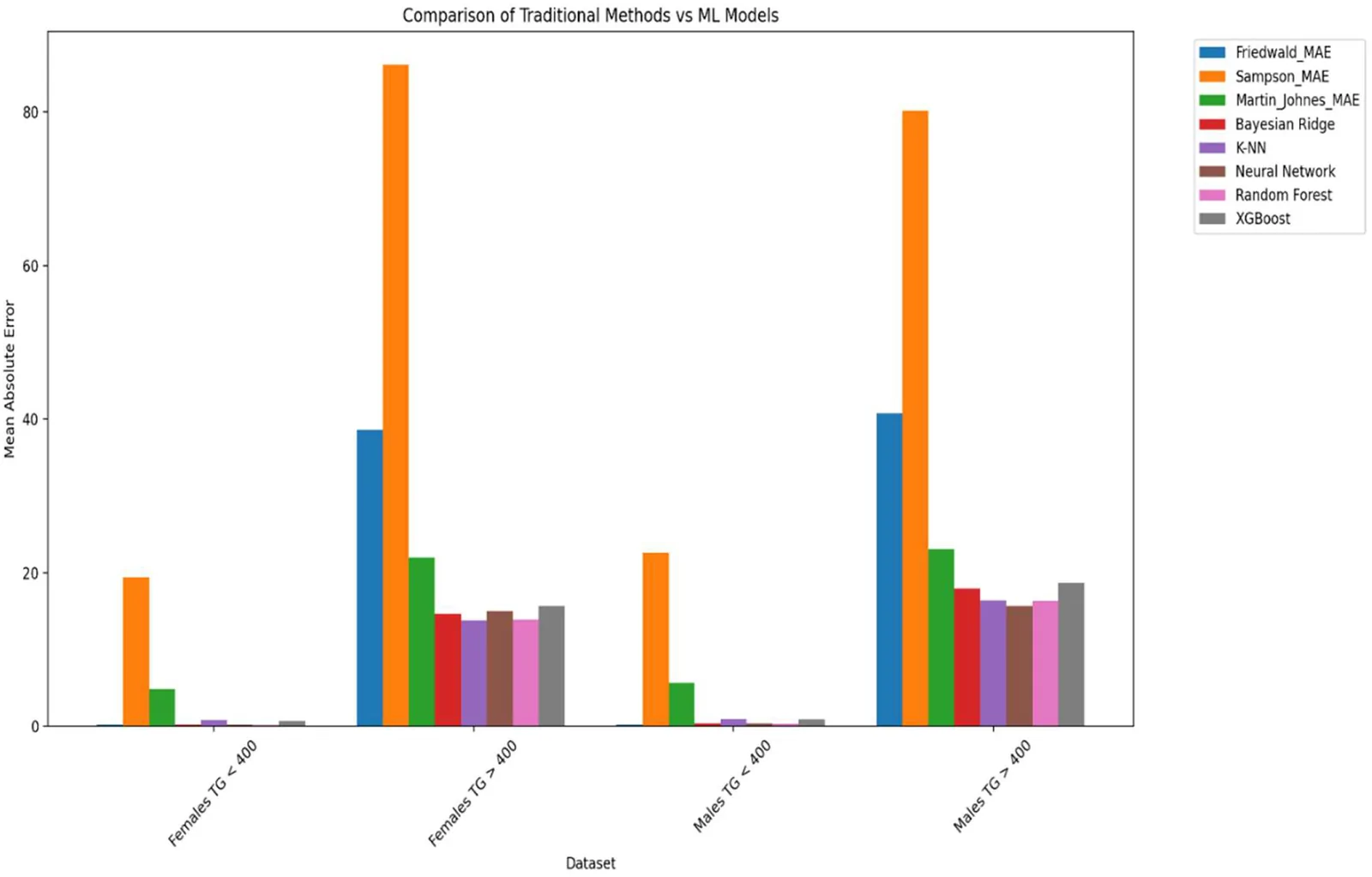
MAE for each ML model across different patient subgroups.

Fig. 4 illustrates the distribution of Mean Absolute Error (MAE) across machine learning models and triglyceride subgroups. In the TG < 400 mg/dL subgroup, MAE values remained low across all models (mean MAE: 0.44 mg/dL in females and 0.50 mg/dL in males), confirming high prediction precision.

In the TG > 400 mg/dL subgroup, MAE increased markedly (mean MAE: 14.62 mg/dL in females and 17 mg/dL in males), reflecting the impact of elevated triglycerides on model accuracy. Differences between models were modest in this subgroup, indicating a generalized reduction in performance rather than model-specific limitations.

**Fig. 5:**
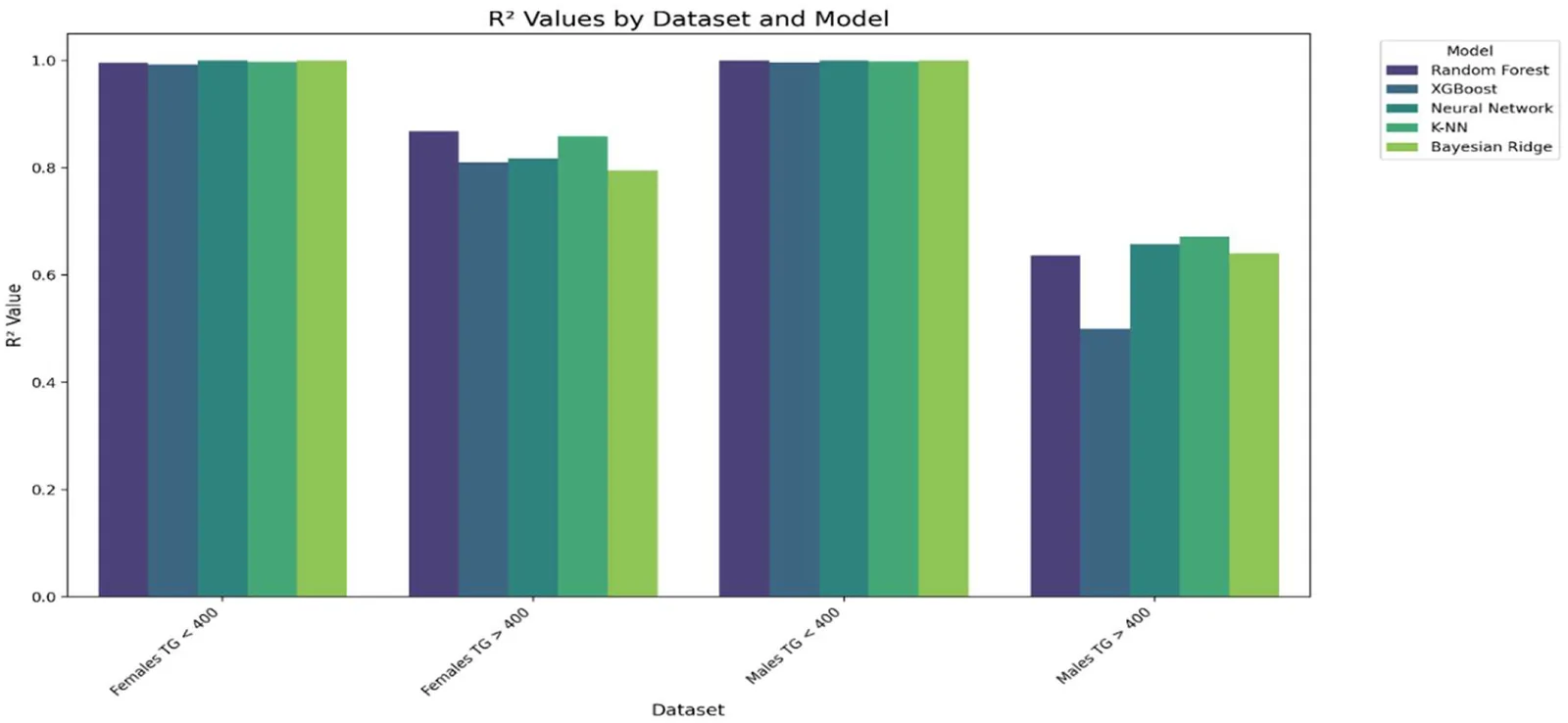
Visual model appearance based on R^2^ for males and females with Triglycerides < 400 mg/dL and > 400 mg/dL.

Fig. 5 provides a visual comparison of model performance based on R² values across sexes and triglyceride strata. In the TG < 400 mg/dL subgroup, all models demonstrated consistently high R^2^ values with minimal inter-model and sex-based variability, indicating stable predictive performance.

However, in the TG > 400 mg/dL subgroup, R^2^ values declined substantially and variability increased across models. This effect was more pronounced in males, where R² values dropped to as low as 0.500 (XGBoost), compared to 0.795–0.868 in females. These findings suggest sex-related differences in model robustness under hypertriglyceridemia conditions.^9^^,^^11^, ^12^, ^13^, ^14^

**Fig. 6:**
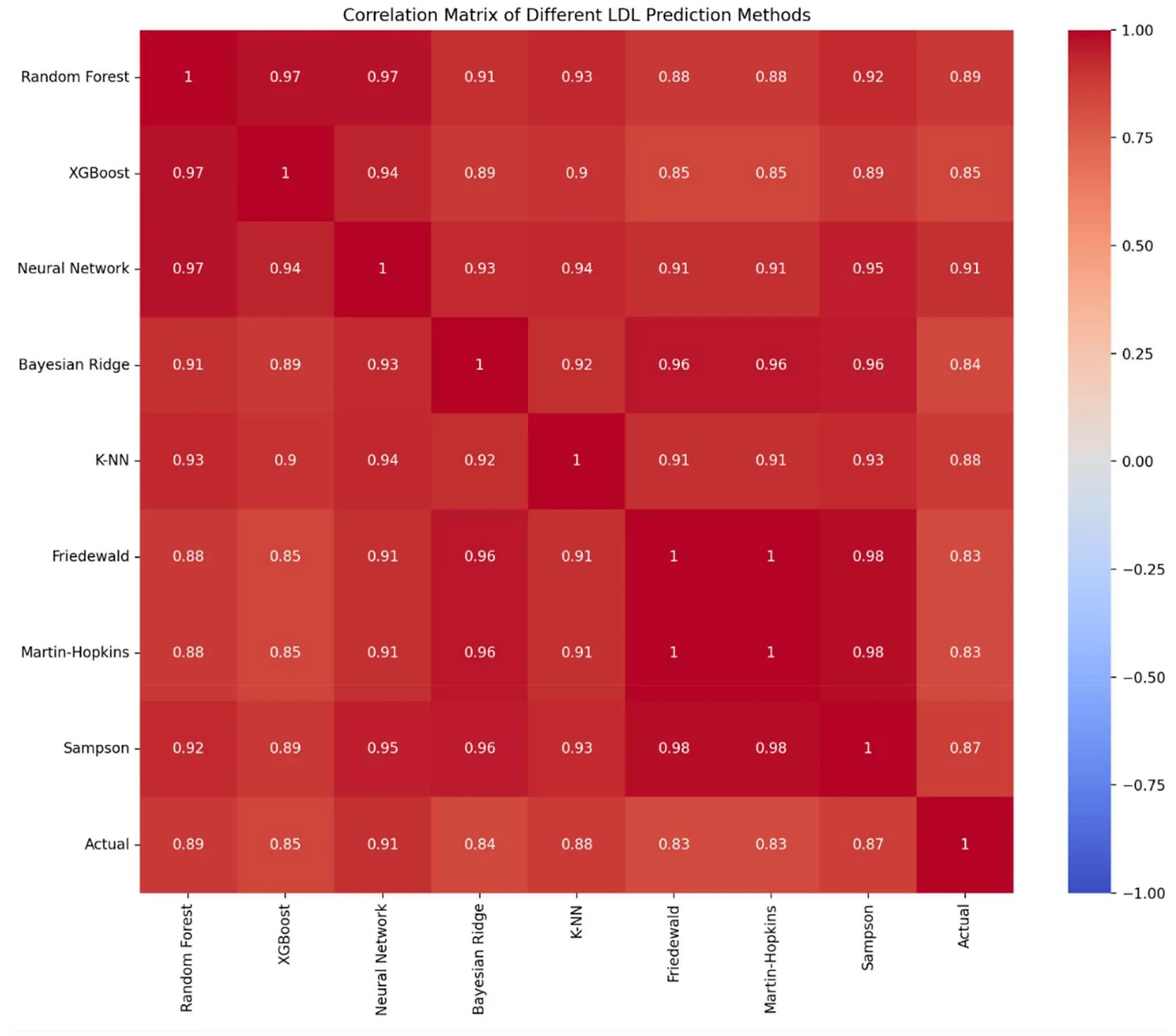
Heatmap of correlation between LDL-C estimation methods (Axis labels represent each LDL-C estimation method (Direct LDL-C, Friedewald, Martin-Hopkins, Sampson, and ML-predicted LDL-C). The color scale legend indicates the correlation coefficient (*r*), where darker color intensities reflect stronger positive correlations.

Fig. 6 presents a correlation heatmap comparing directly measured LDL-C, conventional equations (Friedewald, Martin–Hopkins, Sampson), and machine learning–predicted LDL-C. In the TG < 400 mg/dL subgroup, strong positive correlations were observed across all methods (visually represented by high-intensity color patterns), indicating substantial agreement.

In contrast, the TG > 400 mg/dL subgroup demonstrated weaker and more heterogeneous correlations, reflecting diminished concordance between estimation methods. These findings are supported by Cohen’s kappa analysis, which showed substantial agreement in TG < 400 mg/dL (κ = 0.78–0.89) but only moderate agreement in TG > 400 mg/dL (κ = 0.42–0.55).

Supplementary Fig. S1 presents a heatmap illustrating the correlation between LDL-C estimation methods derived from traditional equations (Friedewald, Martin-Hopkins, and Sampson). Strong positive correlations were observed among all conventional equations, indicating consistent agreement in LDL-C estimation under standard conditions. The highest correlations were observed between Martin–Hopkins and Sampson equations, reflecting their similar methodological refinement compared to the Friedewald equation.

### Classification performance (confusion matrix analysis)

LDL-C results were classified according to the National Cholesterol Education Program Adult Treatment Panel III (NCEP ATP III) guidelines into five categories: optimal (< 100 mg/dL), near optimal (100–129 mg/dL), borderline high (130–159 mg/dL), high (160–189 mg/dL), and very high (≥ 190 mg/dL).

Confusion matrix analysis was performed to evaluate classification accuracy according to NCEP categories.

### Triglycerides > 400 mg/dL subgroup

Supplementary Table S1 shows confusion matrices comparing machine learning predictions with directly measured LDL-C. Across all models, correct classifications were primarily concentrated along the diagonal; however, substantial misclassification was observed, particularly in intermediate LDL-C categories (70–159 mg/dL).

Random Forest demonstrated relatively better classification performance, with higher correct classification counts (e.g., 27 correctly classified cases in the < 70 mg/dL category). In contrast, k-NN, Neural Network, and Bayesian Ridge models showed greater dispersion of predictions into adjacent categories, indicating reduced classification precision.

Overall, the TG > 400 mg/dL subgroup showed moderate agreement with frequent category shifts, reflecting reduced reliability of LDL-C estimation in hypertriglyceridemia.

### Triglycerides < 400 mg/dL subgroup

Supplementary Table S2 shows confusion matrices comparing machine learning predictions with Friedewald-estimated LDL-C values. A strong concentration of observations along the diagonal was observed across all models, indicating high concordance with Friedewald classification.

Random Forest demonstrated particularly high classification agreement (e.g., 3653 correctly classified cases in the < 70 mg/dL category), with minimal misclassification. Similar patterns were observed for Neural Network, k-NN, and Bayesian Ridge models, with misclassification largely limited to adjacent LDL-C categories near clinical thresholds.

These findings indicate substantial classification agreement in TG < 400 mg/dL, consistent with the high statistical performance observed in this subgroup.

## Discussion

In this retrospective study of lipid profile data from Dubai Health, the authors assessed the performance of ML models and various traditional LDL-C estimation methods across TG levels (< 400 mg/dL vs. > 400 mg/dL). The key findings were: ML models (especially Random Forest) provided very high statistical agreement with the routine LDL-C comparator in the TG < 400 mg/dL subgroup^13^^,^^15^^,^^16^; while performance dropped in TG > 400 mg/dL, particularly for males; and traditional formulas showed good but lower concordance in higher TG groups.

Additionally, the use of a single triglyceride threshold (400 mg/dL) may have obscured more nuanced performance differences across intermediate triglyceride ranges. More granular stratification (e.g., < 150 mg/dL, 150–399 mg/dL, ≥ 400 mg/dL) may provide further insight into model behavior and should be explored in future studies.

The machine learning models evaluated in this study were developed to optimize predictive agreement with routinely reported LDL-C values rather than to provide mechanistic insight into lipid metabolism. Consequently, these models function as statistical estimators based on mathematical relationships among total cholesterol, HDL-C, and triglycerides. Formal model interpretability analyses, such as feature importance ranking or SHAP-based explanations, were not performed. This limits assessment of biological plausibility and transparency of model behavior, and future studies should incorporate interpretability frameworks to better align model predictions with established lipid physiology.

Moreover, in literature,^1^ evaluating LDL-C estimation methods have largely focused on comparisons between conventional equations, often within narrowly defined lipid ranges or specific clinical subgroups. While several investigations have demonstrated improved performance of the Martin-Hopkins or Sampson equations over Friedewald estimation, particularly at moderate triglyceride levels, inconsistencies persist in hypertriglyceridemia populations and across different laboratory platforms. In this context, the present study contributes by examining both conventional equations and multiple machine learning approaches within a large, real-world laboratory dataset. Unlike prior studies that relied on smaller or highly selected cohorts, this analysis highlights those apparent gains in statistical performance, particularly in low triglyceride strata, do not uniformly translate to robust performance under conditions of lipid heterogeneity. Importantly, the observed decline in model performance at triglyceride levels > 400 mg/dL underscores a persistent knowledge gap in reliable LDL-C estimation for hypertriglyceridemia patients, which remains inadequately addressed by both traditional equations and data-driven models The present results echo this: in TG > 400 mg/dL subgroups, While several machine learning models demonstrated higher statistical performance metrics than conventional equations when benchmarked against directly measured LDL-C, these findings represent relative agreement with a routinely used clinical assay rather than superiority against a true reference standard. Accordingly, differences in R² and MAE should be interpreted as comparative performance within this analytical framework rather than definitive evidence of methodological superiority. Likewise, another investigation revealed that Martin/Hopkins outperformed Friedewald and Sampson in terms of agreement, particularly at moderate to high TG levels; however, there was significant bias in extreme levels.^17^ These results are also in alignment with the present results that, as TG rises, standard formulas become unreliable, and alternative or ML-based estimators may be useful.

Similarly, in diabetic patient cohorts, Martin/Hopkins and Sampson equations outperformed Friedewald, even with lower accuracy at TG > 400 mg/dL.^18^ The present analysis verifies the differences: for TG < 400 mg/dL, Friedewald and Martin/Hopkins are acceptable, whereas ML techniques provide lower error and larger explained variance. In TG > 400 mg/dL, traditional equations lag more.

Studies on very low LDL-C (e.g., < 70 mg/dL or < 40 mg/dL) indicate that Friedewald underestimates, potentially misclassifying risk. The research demonstrated how novel estimating methods reduce underestimation.^19^ The confusion-matrix results (Figs. S1 and S2) reveal that misclassifications occur in borderline and near-optimal categories, which is consistent with previous research results with traditional formulas are less accurate around treatment thresholds and when LDL-C is low, or TG is high.

### Implementation and validation considerations

The study’s findings come from a large, diverse population within a single healthcare system in Dubai. Although the sample size strengthens internal validity, the results may not be directly generalizable to regions with different demographics, healthcare structures, laboratory methods, or lipid testing practices. Broader application should therefore be cautious, and external validation in independent populations is required before wider clinical adoption.

For clinical implementation, integrating the machine-learning LDL-C estimation model into laboratory information systems would require thorough method validation, including checks for accuracy, precision, reportable ranges, and comparison with direct LDL-C assays. Successful adoption would also depend on clinician education ‒ especially for interpreting results in patients with high triglycerides ‒ and establishing strong model governance, including regular performance monitoring, re-validation, and documentation of model updates to ensure ongoing safety and reliability in routine practice.

In addition, model governance procedures, such as periodic performance monitoring, re-validation, and documentation of model updates, would be necessary to ensure safe and reliable use within routine clinical practice. In current clinical practice, machine learning-based LDL-C estimation should be considered complementary and investigational rather than a replacement for validated equations or direct measurement.

## Conclusion

Among the evaluated machine learning approaches, performance varied across triglyceride subgroups, with no single model demonstrating uniformly robust performance across all triglyceride ranges. In summary, machine learning models demonstrated improved agreement with directly measured LDL-C under specific triglyceride conditions, particularly in TG < 400 mg/dL. However, reduced robustness in hypertriglyceridemia and the absence of external validation limit immediate clinical applicability. Further multicenter validation and interpretability analyses are required before ML-based LDL-C estimation can be considered for routine clinical use.

## Ethics approval and consent to participate

The study was approved by the Ethics Committee of Mohammed Bin Rashid University of Medicine and Health Sciences (MBRU), Dubai Health MBRU-IRB-2025-20. The ethics approval explicitly permitted waiver of informed consent because the study involved retrospective analysis of fully anonymized laboratory data with no direct patient identifiers.

## Consent for publication

Not applicable.

## Authors’ contributions

All authors contributed to the conception, design, data analysis, and manuscript preparation. All authors read and approved the final manuscript.

## Funding

This research received no specific grant from any funding agency in the public, commercial, or not-for-profit sectors.

## Conflicts of interest

The authors declare no conflicts of interest.

## Data Availability

The datasets generated and/or analyzed during the current study are available from the corresponding author upon reasonable request.

## Appendix Supplementary materials

Image, application 1
