# Supplementary material for: Comparative evaluation of LDL-cholesterol estimation using machine learning algorithms and conventional equations in a large middle eastern cohort
Source: Clinics (Sao Paulo). 2026 Sep 8;81:101068. doi: 10.1016/j.clinsp.2026.101068 (PMC13573710; doi:10.1016/j.clinsp.2026.101068)
Supplement: Supplementary file 1 [file mmc1.docx]

**CLINICS-D-25-02060_ Supplementary Material**

**Supplementary File**

**Table S1** Confusion matrix for triglycerides > 400 mg/dL for machine learning model verses direct LDL Cholesterol.

| **Confusion matrix** | **Random Forest < 70** | **Random Forest 70‒99** | **Random Forest 100‒129** | **Random Forest 130‒159** | **Random Forest 160‒189** | **Random Forest ≥ 190** |
| --- | --- | --- | --- | --- | --- | --- |
| Direct LDL cholesterol < 70 mg/dL | 27 | 4 | 1 | 0 | 0 | 0 |
| Direct LDL cholesterol 70‒99 mg/dL | 6 | 18 | 8 | 2 | 0 | 0 |
| Direct LDL cholesterol 100–129 mg/dL | 0 | 15 | 29 | 13 | 2 | 0 |
| Direct LDL cholesterol 130–159 mg/dL | 0 | 2 | 16 | 24 | 3 | 1 |
| Direct LDL cholesterol 160–189 mg/dL | 0 | 0 | 2 | 8 | 16 | 2 |
| Direct LDL cholesterol ≥ 190 mg/dL | 0 | 0 | 1 | 2 | 2 | 6 |
|  | **kNN < 70** | **kNN 70‒99** | **kNN 100‒129** | **kNN 130-159** | **kNN 160‒189** | **kNN ≥190** |
| Direct LDL cholesterol < 70 mg/dL | 17 | 13 | 2 | 0 | 0 | 0 |
| Direct LDL cholesterol 70–99 mg/dL | 4 | 18 | 11 | 1 | 0 | 0 |
| Direct LDL cholesterol 100–129 mg/dL | 0 | 15 | 32 | 12 | 0 | 0 |
| Direct LDL cholesterol 130–159 mg/dL | 0 | 2 | 15 | 26 | 2 | 1 |
| Direct LDL cholesterol 160–189 mg/dL | 0 | 0 | 2 | 11 | 13 | 2 |
| Direct LDL cholesterol ≥ 190 mg/dL | 0 | 0 | 0 | 5 | 1 | 5 |
|  | **Bayesian Ridge < 70** | **Bayesian Ridge 70‒99** | **Bayesian Ridge 100 ≥ 129** | **Bayesian Ridge 130‒159** | **Bayesian Ridge 160‒189** | **Bayesian Ridge ≥ 190** |
| Direct LDL cholesterol < 70 mg/dL | 18 | 11 | 2 | 0 | 0 | 0 |
| Direct LDL cholesterol 70–99 mg/dL | 4 | 16 | 13 | 1 | 0 | 0 |
| Direct LDL cholesterol 100–129 mg/dL | 1 | 11 | 39 | 8 | 0 | 0 |
| Direct LDL cholesterol 130–159 mg/dL | 0 | 2 | 21 | 20 | 1 | 2 |
| Direct LDL cholesterol 160–189 mg/dL | 0 | 0 | 3 | 15 | 9 | 1 |
| Direct LDL cholesterol ≥ 190 mg/dL | 0 | 0 | 0 | 4 | 5 | 2 |
|  | **Neural Net < 70** | **Neural Net 70‒99** | **Neural Net 100‒129** | **Neural Net 130‒159** | **Neural Net 160‒189** | **Neural Net ≥ 190** |
| Direct LDL cholesterol < 70 mg/dL | 23 | 9 | 0 | 0 | 0 | 0 |
| Direct LDL cholesterol 70–99 mg/dL | 7 | 16 | 10 | 1 | 0 | 0 |
| Direct LDL cholesterol 100–129 mg/dL | 0 | 15 | 32 | 11 | 1 | 0 |
| Direct LDL cholesterol 130–159 mg/dL | 0 | 1 | 18 | 25 | 1 | 1 |
| Direct LDL cholesterol 160–189 mg/dL | 0 | 0 | 3 | 8 | 15 | 2 |
| Direct LDL cholesterol ≥ 190 mg/dL | 0 | 0 | 0 | 2 | 4 | 5 |

**Table S2** Confusion matrix for triglycerides < 400 mg/dL for machine learning model verses direct LDL Cholesterol.

| **Confusion Matrix** | **Bayesian Ridge < 70** | **Bayesian Ridge 70‒99** | **Bayesian Ridge 100‒129** | **Bayesian Ridge 130‒159** | **Bayesian Ridge 160‒189** | **Bayesian Ridge ≥ 190** |
| --- | --- | --- | --- | --- | --- | --- |
| Freidwald LDL Cholesterol < 70 mg/dL | 1463 | 2566 | 116 | 1 | 0 | 0 |
| Freidwald LDL Cholesterol 70‒99 mg/dL | 830 | 5111 | 855 | 5 | 1 | 0 |
| Freidwald LDL Cholesterol 100‒129 mg/dL | 68 | 4896 | 1903 | 55 | 9 | 0 |
| Freidwald LDL Cholesterol 130‒159 mg/dL | 0 | 1645 | 2218 | 171 | 36 | 0 |
| Freidwald LDL Cholesterol 160‒189 mg/dL | 0 | 73 | 1063 | 234 | 32 | 0 |
| Freidwald LDL Cholesterol ≥ 190mg/dL | 0 | 0 | 181 | 230 | 42 | 8 |
|  | **Random Forest < 70** | **Random Forest 70–99** | **Random Forest 100–129** | **Random Forest 130–159** | **Random Forest 160–189** | **Random Forest ≥190** |
| Freidwald LDL Cholesterol < 70 mg/dL | 3653 | 480 | 13 | 0 | 0 | 0 |
| Freidwald LDL Cholesterol 70–99 mg/dL | 1221 | 5261 | 315 | 5 | 0 | 0 |
| Freidwald LDL Cholesterol 100–129mg/dL | 0 | 3278 | 3499 | 154 | 0 | 0 |
| Freidwald LDL Cholesterol 130–159 mg/dL | 0 | 1 | 2816 | 1224 | 29 | 0 |
| Freidwald LDL Cholesterol 160–189 mg/dL | 0 | 0 | 11 | 1210 | 179 | 2 |
| Freidwald LDL Cholesterol ≥ 190mg/dL | 0 | 0 | 0 | 30 | 306 | 125 |
|  | **Neural Net < 70** | **Neural Net 70–99** | **Neural Net 100–129** | **Neural Net 130–159** | **Neural Net 160–189** | **Neural Net ≥ 190** |
| Freidwald LDL Cholesterol < 70 mg/dL | 3650 | 481 | 15 | 0 | 0 | 0 |
| Freidwald LDL Cholesterol 70–99 mg/dL | 1221 | 5254 | 323 | 4 | 0 | 0 |
| Freidwald LDL Cholesterol 100–129 mg/dL | 0 | 3281 | 3502 | 148 | 0 | 0 |
| Freidwald LDL Cholesterol 130–159 mg/dL | 0 | 1 | 2931 | 1110 | 28 | 0 |
| Freidwald LDL Cholesterol 160–189 mg/dL | 0 | 0 | 25 | 1212 | 163 | 2 |
| Freidwald LDL Cholesterol ≥ 190mg/dL | 0 | 0 | 0 | 46 | 298 | 117 |
|  | **kNN < 70** | **kNN 70–99** | **kNN 100–129** | **kNN 130–159** | **kNN 160–189** | **kNN ≥190** |
| Freidwald LDL Cholesterol < 70 mg/dL | 3623 | 510 | 13 | 0 | 0 | 0 |
| Freidwald LDL Cholesterol 70‒99 mg/dL | 1164 | 5301 | 333 | 4 | 0 | 0 |
| Freidwald LDL Cholesterol 100–129mg/dL | 0 | 3181 | 3605 | 145 | 0 | 0 |
| Freidwald LDL Cholesterol 130–159mg/dL | 0 | 1 | 2885 | 1157 | 27 | 0 |
| Freidwald LDL Cholesterol 160–189 mg/dL | 0 | 0 | 18 | 1212 | 172 | 0 |
| Freidwald LDL Cholesterol ≥ 190mg/dL | 0 | 0 | 0 | 39 | 308 | 114 |

**Figure S1** Heatmap of correlation between LDL-C estimation methods based on traditional equations of LDL cholesterol.
